# Agonist-independent Gα_z_ activity negatively regulates beta-cell compensation in a diet-induced obesity model of type 2 diabetes

**DOI:** 10.1074/jbc.RA120.015585

**Published:** 2020-11-24

**Authors:** Michael D. Schaid, Cara L. Green, Darby C. Peter, Shannon J. Gallagher, Erin Guthery, Kathryn A. Carbajal, Jeffrey M. Harrington, Grant M. Kelly, Austin Reuter, Molly L. Wehner, Allison L. Brill, Joshua C. Neuman, Dudley W. Lamming, Michelle E. Kimple

**Affiliations:** 1 Research Service, William S. Middleton Memorial Veterans Hospital, Madison, Wisconsin, USA; 2 Interdepartmental Graduate Program in Nutritional Sciences, College of Agriculture and Life Sciences, University of Wisconsin-Madison, Madison, Wisconsin, USA; 3 Division of Endocrinology, Department of Medicine, University of Wisconsin-Madison School of Medicine and Public Health, Madison, Wisconsin, USA; 4 Department of Cell and Regenerative Biology, University of Wisconsin- Madison School of Medicine and Public Health, Madison, Wisconsin, USA

**Keywords:** insulin secretion, insulin resistance, diabetes, cAMP, G protein, G protein–coupled receptor (GPCR), cell signaling, βKO, beta-cell–specific Gαz-null, AUC, area under the curve, BCA, bicinchoninic acid, B6J, C57BL/6J, B6N, C57BL/6N, CD, control diet, EIA, enzyme immunoassay, EP3, prostaglandin EP3 receptor, Ex4, exendin-4, FBKO, Gαz full-body KO mice on the B6N background, Gα_z_, G protein alpha-subunit, GLP1R, glucagon-like peptide 1 receptor, GPCRs, G protein–coupled receptors, GSIS, glucose-stimulated insulin secretion, HA-EP3γ, hemagglutinin-tagged EP3γ, HFD, high-fat diet, HRP, horseradish peroxidase, IBMX, 3-isobutyl-1-methylxanthine, ITTs, insulin tolerance tests, KEGG, Kyoto Encyclopedia of Genes and Genomes, KRBB, Krebs-Ringer bicarbonate buffer, OGTTs, oral glucose tolerance tests, PGE_2_, prostaglandin E_2_, qRT-PCR, quantitative real-time PCR, RIA, radioimmunoassay, SDE, significantly differentially expressed, T2D, type 2 diabetes

## Abstract

The inhibitory G protein alpha-subunit (Gα_z_) is an important modulator of beta-cell function. Full-body Gα_z_-null mice are protected from hyperglycemia and glucose intolerance after long-term high-fat diet (HFD) feeding. In this study, at a time point in the feeding regimen where WT mice are only mildly glucose intolerant, transcriptomics analyses reveal islets from HFD-fed Gα_z_ KO mice have a dramatically altered gene expression pattern as compared with WT HFD-fed mice, with entire gene pathways not only being more strongly upregulated or downregulated *versus* control-diet fed groups but actually reversed in direction. Genes involved in the “pancreatic secretion” pathway are the most strongly differentially regulated: a finding that correlates with enhanced islet insulin secretion and decreased glucagon secretion at the study end. The protection of Gα_z_-null mice from HFD-induced diabetes is beta-cell autonomous, as beta cell–specific Gα_z_-null mice phenocopy the full-body KOs. The glucose-stimulated and incretin-potentiated insulin secretion response of islets from HFD-fed beta cell–specific Gα_z_-null mice is significantly improved as compared with islets from HFD-fed WT controls, which, along with no impact of Gα_z_ loss or HFD feeding on beta-cell proliferation or surrogates of beta-cell mass, supports a secretion-specific mechanism. Gα_z_ is coupled to the prostaglandin EP3 receptor in pancreatic beta cells. We confirm the EP3γ splice variant has both constitutive and agonist-sensitive activity to inhibit cAMP production and downstream beta-cell function, with both activities being dependent on the presence of beta-cell Gα_z_.

A crucial factor in the development of type 2 diabetes (T2D) is failure of beta cells to adapt their secretory phenotype and/or mount a compensatory increase in mass in the face of peripheral insulin resistance (1). In the beta cell, cAMP is a critical second messenger essential for proper beta-cell function (2). Synthesis of cAMP is primarily regulated by G protein–coupled receptors (GPCRs) and their associated heterotrimeric G protein complex. GPCR action and cAMP signaling are essential in glucose-stimulated insulin secretion (GSIS) response and ability of the beta cell to compensate under stress (2). A variety of T2D therapeutics bind to and activate the glucagon-like peptide 1 receptor (GLP1R), which acts through G_s_-subfamily proteins to increase the catalytic activity of adenylyl cyclase and downstream cAMP production (3). Conversely, inhibition of adenylyl cyclase by G proteins of the G_i/o_ subfamily reduces cAMP levels and negatively regulates beta-cell function (4). Yet, no T2D drug that targets inhibitory GPCRs has ever been identified for widespread clinical use.

We have previously demonstrated a role for the alpha subunit of the unique G_i/o_ subfamily member, G_z_ (G protein alpha-subunit [Gα_z_]), in beta-cell biology (5, 6, 7, 8, 9). Islets from transgenic KO mice lacking Gα_z_ throughout their body have elevated pancreatic islet cAMP levels and secrete more insulin in response to glucose, accelerating glucose clearance (7). C57BL/6N (B6N) mice subjected to extended high-fat diet (HFD) feeding become morbidly obese and insulin resistant, with fasting hyperglycemia and glucose intolerance mimicking T2D. Loss of Gα_z_ throughout the body is sufficient to protect from these aspects the phenotype, even in the face of obesity and insulin resistance mirroring WT HFD controls (8). Coupled with the extremely limited tissue distribution of Gα_z_ protein (found only in the brain, platelets, retina, and pancreatic islets (10, 11)), this phenotype fits best with a beta cell–centric model.

Previous work from our laboratory has implicated prostaglandin EP3 receptor (EP3), whose most abundant native ligand is the arachidonic acid metabolite, prostaglandin E_2_ (PGE_2_), in mediating the inhibitory effects of Gα_z_ on islet cAMP production and GSIS (8, 9). Yet, the primary effects of EP3 on beta-cell biology have been suggested to be on proliferation, not function (12, 13). Furthermore, extra-islet EP3 signaling is crucial in maintaining glucose homeostasis (13, 14, 15, 16). Therefore, many questions remain as to the precise molecular and cellular mechanisms by which Gα_z_ loss protects from hyperglycemia and glucose intolerance and whether beta-cell EP3 is actually a viable target for T2D therapeutics. In this study, we use islet transcriptomic analysis and beta-cell–specific Gα_z_-null (βKO) mice in the C57BL/6J (B6J) background to demonstrate, in the absence of alterations in the overall EP3 expression or activity of agonist-dependent EP3 variants, Gα_z_ has differential effects on beta-cell function and mass depending on the pathophysiological context of the model.

## Results

### An interplay between Gα_z_ loss and HFD feeding dramatically alters islet secretory pathway gene expression

**Figure 1:**
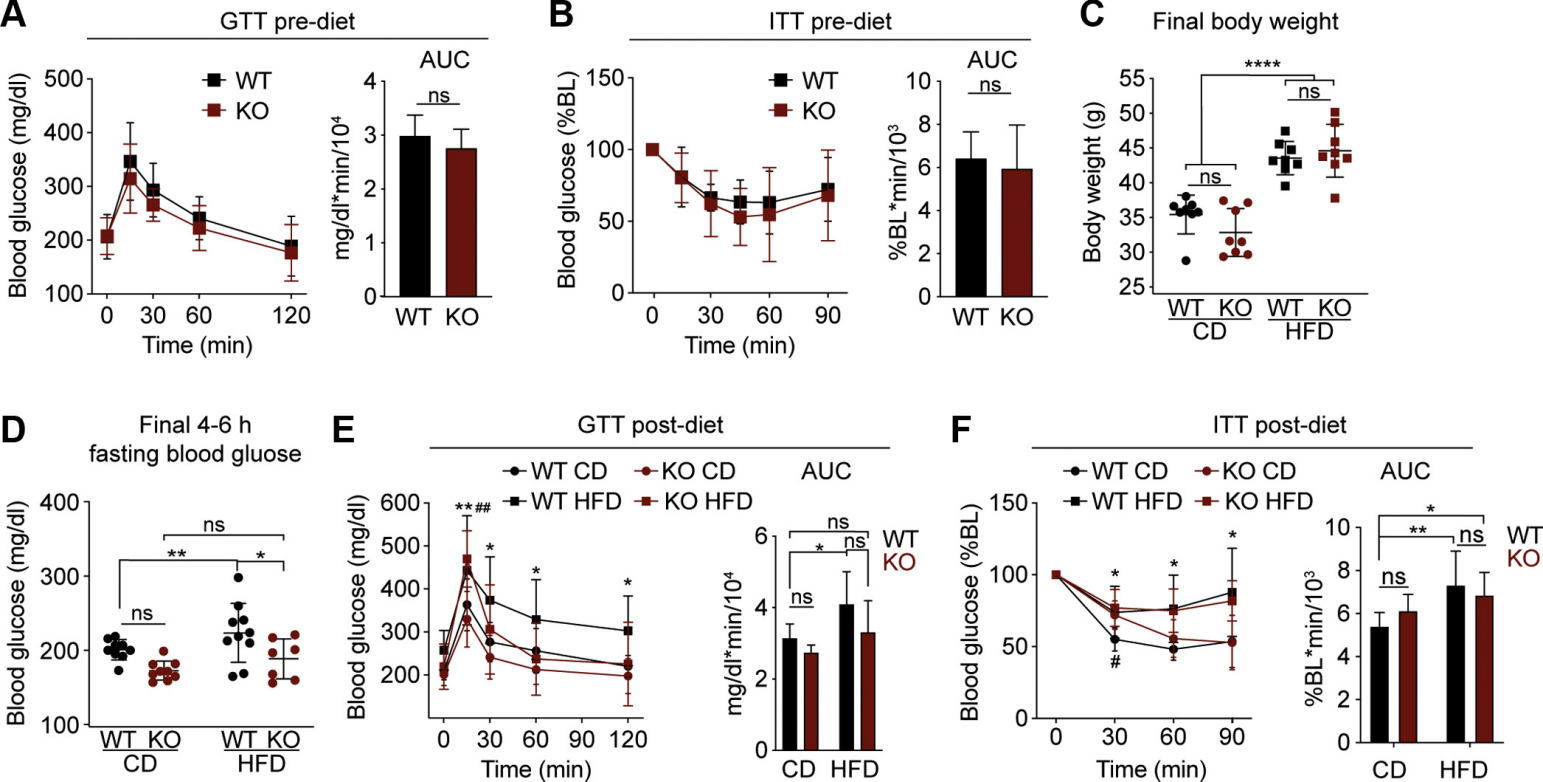
**Sixteen-week HFD feeding of C57BL/6N mice results in mild hyperglycemia and glucose intolerance ameliorated by loss of Gα**_**z**_**independent of effects on insulin sensitivity.***A*–*B*, baseline metabolic phenotypes of 11-week-old chow-fed WT or KO mice. *A*, oral glucose tolerance (1 g/kg) as represented by glucose excursion (*left*) or AUC from zero (*right*). Sixteen mice/group. *B*, IP insulin tolerance (0.75 U/kg) as represented by glucose excursion normalized to baseline (*left*) or AUC of the normalized data from zero (*right*). N=9 to 12 mice/group. *C*–*F*, metabolic phenotypes of 27-week-old WT and KO mice after 16 weeks of control diet (CD) or high-fat diet (HFD) feeding. N = 7 to 10 mice/group. *C*, body weights. *D*, fasting blood glucose levels of 4 to 6 h. *E*, oral glucose tolerance (1 g/kg) as represented by glucose excursion (*left*) or AUC from zero (*right*). *F*, IP insulin tolerance (0.75 U/kg) as represented by glucose excursion normalized to baseline (*left*) or AUC of the normalized data from zero (*right*). Data represent mean ± SD and were analyzed as described in Experimental procedures. ∗*p* < 0.05; ∗∗*p* < 0.01; ∗∗∗∗*p* < 0.0001. In panel *E*, ##*p* < 0.01 for KO HFD *versus* WT CD at the 15-min time point. AUC, area under the curve; Gα_z_, G protein alpha-subunit; ns, not significant.

We have previously shown that male B6N mice deficient in a protein-coding exon of *Gnaz*, the gene encoding for Gα_z_, are fully protected from hyperglycemia and glucose intolerance, even after up to 26 to 30 weeks of consuming a 45 kcal% fat diet (HFD) starting at 11 weeks of age (8). HFD feeding in Gα_z_ KO mice approximately doubles functional beta-cell mass, independent of any changes in the weight gain, food intake, or insulin resistance as compared with WT HFD-fed controls (8). To determine potential molecular pathways by which Gα_z_ might influence the beta-cell compensation response, we performed a shorter (16-week) diet study—a feeding regimen chosen to limit potential confounding effects of long-term uncontrolled hyperglycemia—and isolated islets for exon array analyses. As with our previous cohort (8), there were no differences in baseline blood glucose or insulin tolerance by genotype (Fig. 1, *A*–*B*). After 16 weeks of control diet (CD) or HFD feeding, both HFD-fed groups were significantly heavier than CD-fed groups, and there were no differences in the body weight by genotype (Fig. 1*C*). The 4- to 6-h fasting blood glucose levels of WT HFD-fed mice were mildly elevated as compared with CD-fed animals (Fig. 1*D*), and Gα_z_ loss essentially ameliorated the mild fasting hyperglycemia and oral glucose intolerance of male B6N mice after 16 weeks of HFD feeding (Fig. 1*E*). Gα_z_ loss again had little-to-no effect on the development of insulin resistance, whether quantified by two-way ANOVA of the percentage baseline blood glucose curves (Fig. 1*F*, left) or by one-way ANOVA of the area under the curves from Y = 0 (Fig. 1*F*, right).

**Figure 2:**
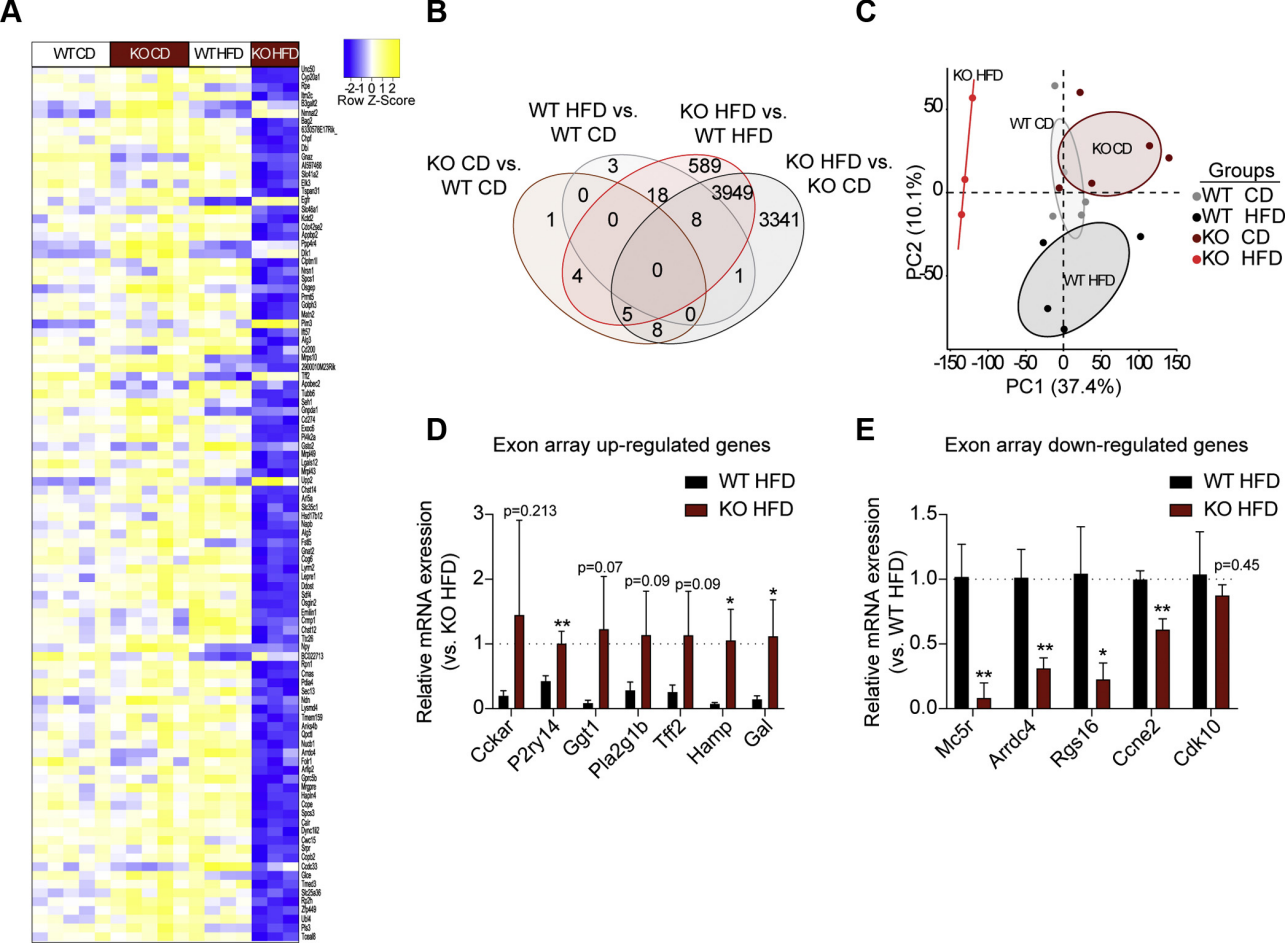
**Sixteen-week high-fat diet (HFD) feeding of Gα**_**z**_**KO mice results in wholesale alterations in gene expression profiles as compared with WT HFD-fed controls.***A*–*C*, the transcriptomics profile of isolated islets from WT and Gα_z_ KO islets after 16-week HFD or control diet (CD) feeding. N = 3 to 5 independent islet preparations/group. *A*, the heatmap indicating the top 100 most variable significantly differentially expressed genes across the four groups (Benjamini–Hochberg–adjusted *p*-value < 0.05). Z-score indicates SDs away from the mean log cpm in either a positive or negative direction. Each column represents a single mouse. *B*, four-set Venn diagram representing the total number of differentially expressed genes between groups. *C*, principal component analysis of microarray data. The numbers are indicative of mouse ID. *D*–*E*, validation of differentially upregulated (*D*) and downregulated (*E*) genes by qRT-PCR. Three independent islet preparations per group. Data represent mean ± SD and were compared within each gene by unpaired *t*-test. *p*-values > 0.05 are labeled. ∗*p* < 0.05; ∗∗*p* < 0.01. AUC, area under the curve; Gα_z_, G protein alpha-subunit; qRT-PCR, quantitative real-time PCR.

Total islet RNA was subjected to exon-array gene chip analysis. Islet gene expression results from 5 WT CD-fed mice, 5 KO CD-fed mice, 4 WT HFD-fed mice, and 3 KO HFD-fed mice passed quality control tests and were used for downstream analysis. Unsupervised hierarchical clustering revealed samples from each group segregated together, with islets from KO HFD-fed mice having the greatest differentiating gene expression profile among the four groups. The top 100 most significantly differentially expressed (SDE) genes across all comparisons are shown in Figure 2*A*, and the gene symbols according to their placement in the dendrogram can be found in Table S1. Of 16,291 expressed genes, 18 genes were SED (adjusted *p*-value < 0.05) between WT and KO CD-fed mice, 30 genes were SDE between WT CD-fed and HFD-fed mice, 4573 genes were SDE between WT and KO HFD-fed mice, and 7312 genes were SDE between KO CD-fed and HFD-fed mice (Fig. 2*B*). We applied a principal component analysis to all expressed genes and found the first two principal components accounted for approximately 50% of the variance in the levels of gene expression (Fig. 2*C*). The complete data set can be found in the NCBI gene expression omnibus database as accession ID GSE154325.

To validate the microarray results by quantitative real-time PCR (qRT-PCR), we selected from among the top 50+ upregulated and downregulated SDE genes between WT HFD and KO HFD (Tables S2 and S3, respectively) based on potential mechanistic relevance to a beta-cell secretion or the replication phenotype. These included genes encoding GPCRs (cholecystokinin A receptor; melanocortin 5 receptor; P2Y purinoceptor 14), regulators of GPCRs or G proteins (arrestin domain containing 4; regulator of G protein signaling 16), secreted peptides (Galanin; hepcidin antimicrobial protein; trefoil factor 2), metabolic enzymes producing secreted or excreted peptides or other factors (gamma-glutamyltransferase 1, phospholipase A2 group 1b), and cell-cycle genes (cyclin E2 [*Ccne2*]; cyclin-dependent kinase 10). The directionality of expression change between the two groups was validated for each gene, with many of these changes being statistically significant (Fig. 2, *D*–*E*). Notably, *Ccne2* and cyclin-dependent kinase 10 were actually lower in islets from KO HFD-fed mice, with *Ccne2* being significantly so (Fig. 2*E*, right bars).

**Figure 3:**
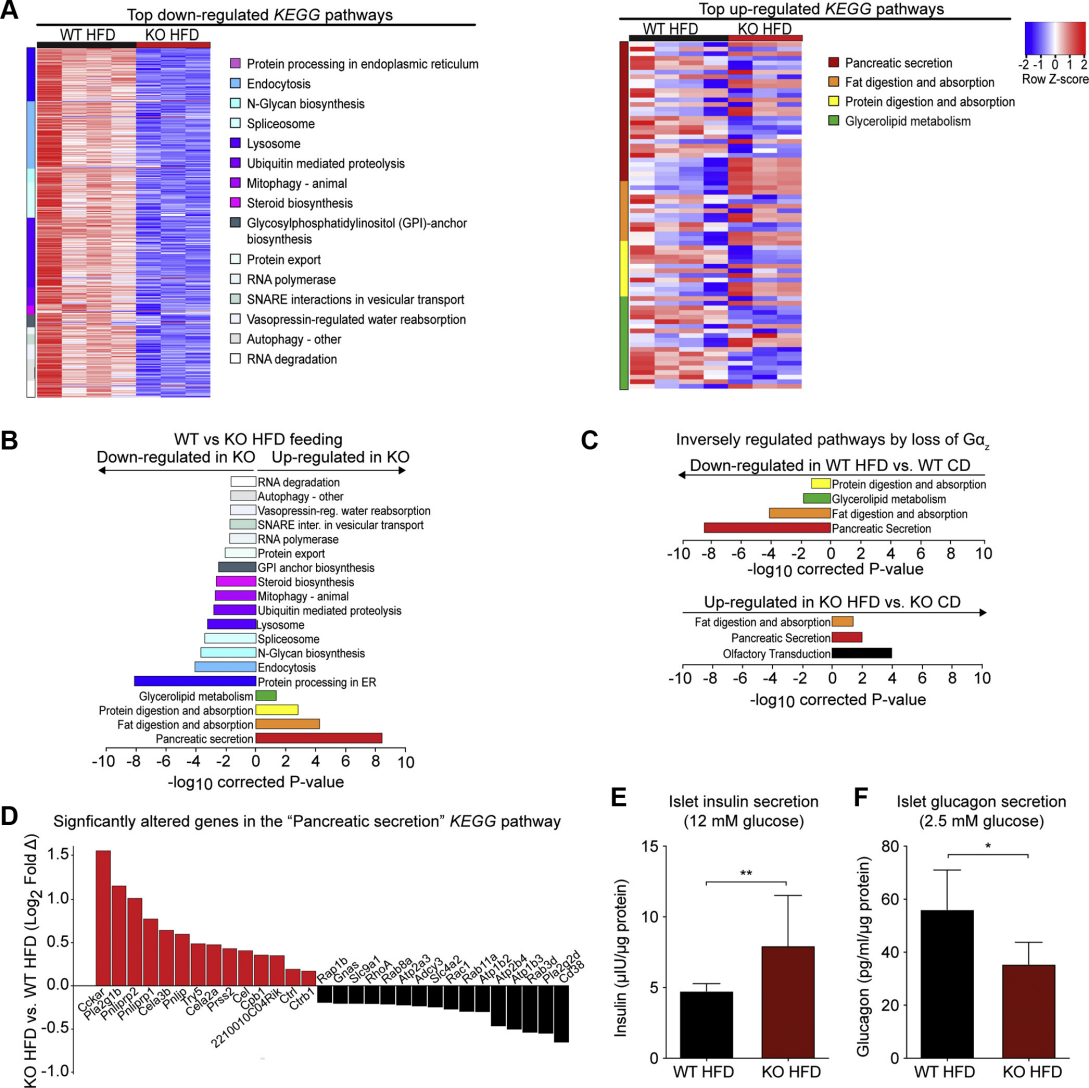
**Sixteen-week HFD feeding of Gα**_**z**_**KO mice significantly alters islet secretion–related gene profiles: an effect that correlates with enhanced insulin secretion at study end.***A*–*D*, transcriptomics profile of isolated islets from WT and Gα_z_ KO islets after 16-week high-fat diet (HFD) or control diet (CD) feeding. N = 3 to 5 independent islet preparations/group. *A*, the heatmap indicating the relative expression of genes involved in the most significantly enriched biological Kyoto Encyclopedia of Genes and Genomes (KEGG) pathways based on genes differentially expressed in islets from HFD-fed WT *versus* KO mice (q < 0.05, false-discovery rate). Genes in more than one significantly enriched KEGG pathway are listed only once and assigned to the most significantly affected pathway. *B*, pathway enrichment analysis of KEGG pathways significantly upregulated or downregulated islets from HFD-fed WT *versus* HFD-fed KO mice. Colors are matched to those of the pathways shown in panel *A*. *C*, pathway enrichment analysis performed as shown in panel *B* showing HFD feeding has an inverse effect on significantly affected pathways in islets from WT *versus* KO mice. Colors are matched to those of the pathways shown in panel *A*. *D*, significantly altered genes in the pancreatic secretion pathway in islets from HFD-fed KO *versus* WT mice. Data were analyzed as described in Experimental procedures. *E*–*F*, insulin secretion (*E*) and glucagon secretion (*F*), both as normalized to total protein, from islets isolated from WT and Gα_z_ KO mice after 25 to 29 weeks of HFD feeding (8). N = 5 to 7 independent islet preparations/group. Data represent mean ± SD and were compared by unpaired *t*-test. ∗*p* < 0.05; ∗∗*p* < 0.01. Gα_z_, G protein alpha-subunit.

We identified pathways in which SDE genes were involved using the Kyoto Encyclopedia of Genes and Genomes (KEGG) database. Although there were no pathways in either analysis that were significantly different in islets from WT *versus* KO CD-fed mice, islets from HFD-fed mice showed significant changes in gene expression across several pathways when compared with diet or genotype controls (Fig. 3*A*). Focusing specifically on KEGG pathways altered between islets of HFD-fed WT *versus* KO mice, several pathways had decreased gene expression in KO mice, including protein-turnover related pathways such as those regulating the proteasome, ribosome biogenesis, RNA polymerase, RNA degradation, protein export, proteolysis, ubiquitin-mediated proteolysis, spliceosome, N-glycan biosynthesis, protein processing in the endoplasmic reticulum, autophagy, mitophagy, and lysosome handling (Fig. 3, *A*–*B* and Table S4). (As nearly all the genes in the “metabolism” and “sphingolipid metabolism” pathways were components of other KEGG pathways, these were excluded in the heat map shown in Fig. 3*A* for clarity). Only four KEGG pathways were upregulated in HFD-fed KO islets *versus* HFD-fed WT islets—glycerolipid metabolism, protein digestion and absorption, fat digestion and absorption, and pancreatic secretion (Fig. 3, *A*–*B* and Table S4). Interestingly, these are the same four pathways significantly downregulated in WT CD *versus* WT HFD islets (Fig. 3*C* and Table S5). KO mice showed the inverse relationship, in that fat digestion and pancreatic secretion, as well as olfactory transduction, were upregulated by HFD feeding (Fig. 3C and Table S6). In all three comparisons, pancreatic secretion was the most significant differentially regulated KEGG pathway in HFD feeding (downadjusted *p*-value = 5.027 × 10^−10^, WT CD *versus* WT HFD; upadjusted *p*-value = 3.71 × 10^−9^, WT HFD *versus* KO HFD; and upadjusted *p*-value = 1.0 × 10^−2^ KO HFD *versus* KO CD). Figure 3*D* shows a diagram of the Log2 fold-change of the SDE genes in the pancreatic secretion pathway in WT HFD *versus* KO HFD, with a number of these as known regulators or effectors of cAMP signaling in the beta cell. These gene expression changes after 16 weeks of HFD feeding are associated with significantly higher *ex vivo* islet insulin secretion after 26 to 30 weeks of HFD feeding (Fig. 3*E*), at the same time that glucagon secretion is lower in islets from HFD-fed KO mice as than in WT mice (Fig. 3*F*). Therefore, the resistance of Gαz KO mice to HFD-induced glucose intolerance appears due to enhanced insulin secretory capacity and not a primary effect of Gαz loss on beta-cell replication. Furthermore, owing to the wholesale shift in pancreatic secretion pathways, many of which are associated with cAMP signaling, it is unlikely any one gene or handful of genes is responsible for this phenotype.

### Beta-Cell loss of Gα_z_ protects against HFD-induced fasting hyperglycemia and glucose intolerance

**Figure 4:**
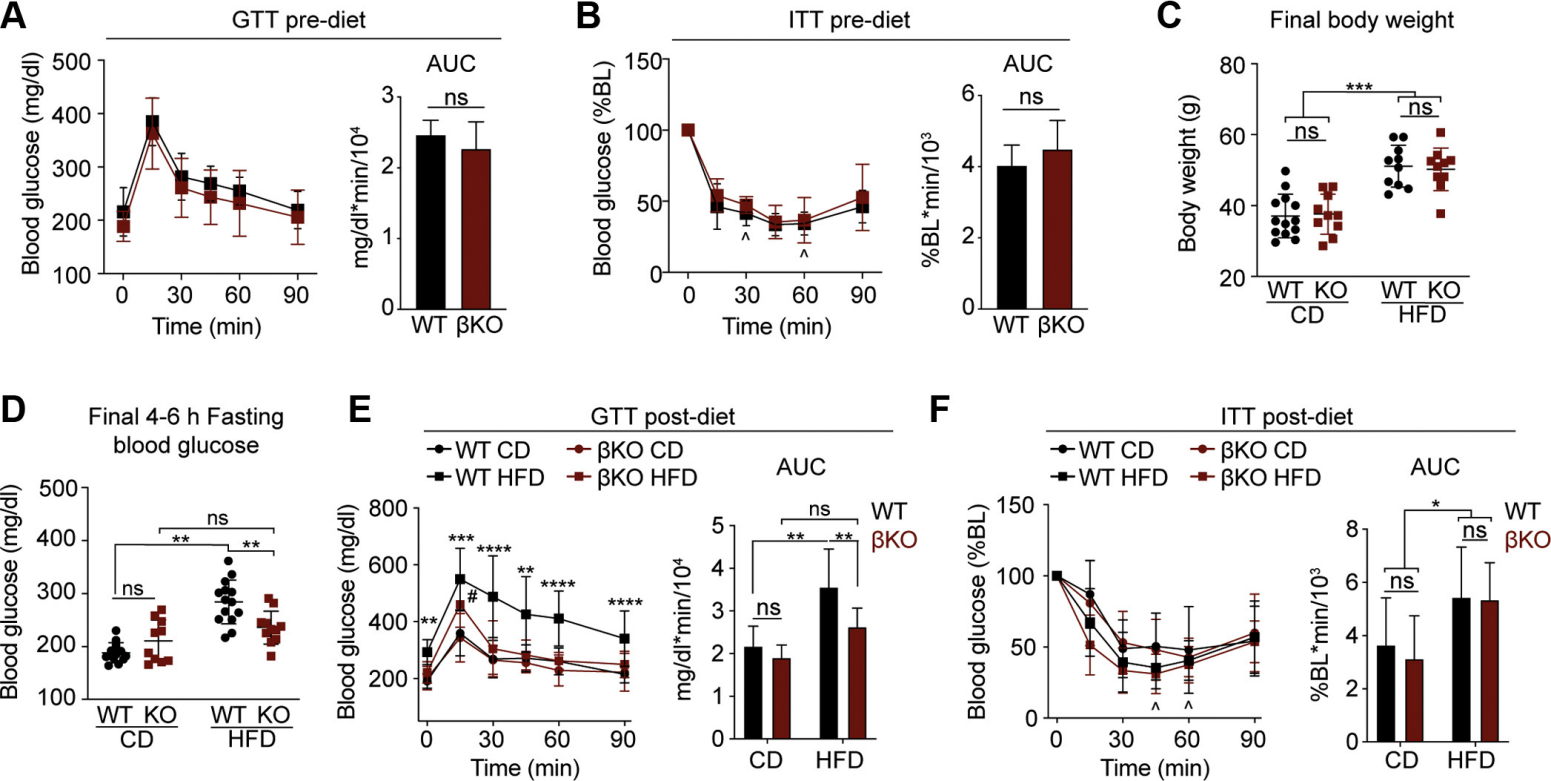
**Beta cell–specific loss of Gα**_**z**_**protects C57BL/6J mice from developing hyperglycemia and glucose intolerance after 28 weeks of HFD feeding independent of effects on insulin sensitivity.***A*–*B*, baseline metabolic phenotypes of 11-week-old chow-fed WT and βKO mice. *A*, oral glucose tolerance (1 g/kg) as represented by glucose excursion (*left*) or AUC from zero (*right*). Twelve to 19 mice/group. *B*, IP insulin tolerance (0.75 U/kg) as represented by glucose excursion normalized to baseline (*left*) or AUC of the normalized data from zero (*right*). N = 9 to 18 mice/group. *C*–*F*, metabolic phenotypes of 39-week-old WT and βKO mice after 28 weeks of control diet (CD) or high-fat diet (HFD) feeding. Ten to 14 mice/group. *C*, body weights. *D*, 4 to 6 h fasting blood glucose levels. *E*, oral glucose tolerance (1 g/kg) as represented by glucose excursion (*left*) or AUC from zero (*right*). *F*, IP insulin tolerance (0.75 U/kg) as represented by glucose excursion normalized to baseline (*left*) or AUC of the normalized data from zero (*right*). Data represent mean ± SD and were analyzed as described in Experimental procedures. ∗*p* < 0.05; ∗∗*p* < 0.01; ∗∗∗*p* < 0.001; ∗∗∗∗*p* < 0.0001. In panel *E*, #*p* < 0.05 for KO HFD *versus* WT CD at the 15-min time point. ˆ in panels *B* and *F* indicate time points in which young and/or CD-fed mice of both genotypes had to be rescued by glucose injection during ITTs (n = 2 of 18 WT and 1 of 9 βKO in panel *B* and 5 of 12 WT and 5 of 10 βKO in panel *F*). AUC, area under the curve; Gα_z_, G protein alpha-subunit; ITT, insulin tolerance tests; ns, not significant.

Gα_z_ protein expression is limited to the brain, retina, platelets, and pancreatic islets (10, 11). To prove the effect of Gα_z_ loss on protection from glucose intolerance is beta cell specific, we repeated the original extended HFD feeding study using a previously generated and validated βKO transgenic line in the B6J background (6). Like the full-body Gα_z_ KO mice in the B6N substrain, 12-week-old βKO had nearly identical glucose and insulin tolerance as their WT controls (Fig. 4, *A*–*B*). After 28 weeks of CD or HFD feeding, both HFD-fed groups were significantly heavier than CD-fed mice, with no differences in weight by genotype (Fig. 4*C*). As with the original B6N study (8), fasting blood glucose and glucose tolerance were severely impacted by HFD feeding in WT B6J mice: effects that were significantly decreased in βKO mice (Fig. 4, *D*–*E*). Yet, in contrast to our previous study, but consistent with prior B6J HFD studies (17, 18), insulin sensitivity was only mildly affected by long-term HFD feeding and did not differ by genotype (Fig. 4*F*). Experimental evidence of the increased insulin sensitivity of lean B6J mice comes from the fact several young mice or CD-fed mice, regardless of the genotype, had to be rescued with glucose injection 30, 45, or 60 min after insulin injection (noted by ˆ in Fig. 4, *B*–*F*). In all of our previous studies in the B6N substrain, we have never had to rescue a mouse because of hypoglycemia during insulin tolerance tests (ITTs) (M.E.K., personal observations).

### Beta-cell loss of Gα_z_ does not enhance beta-cell replication in the insulin-sensitive B6J substrain

**Figure 5:**
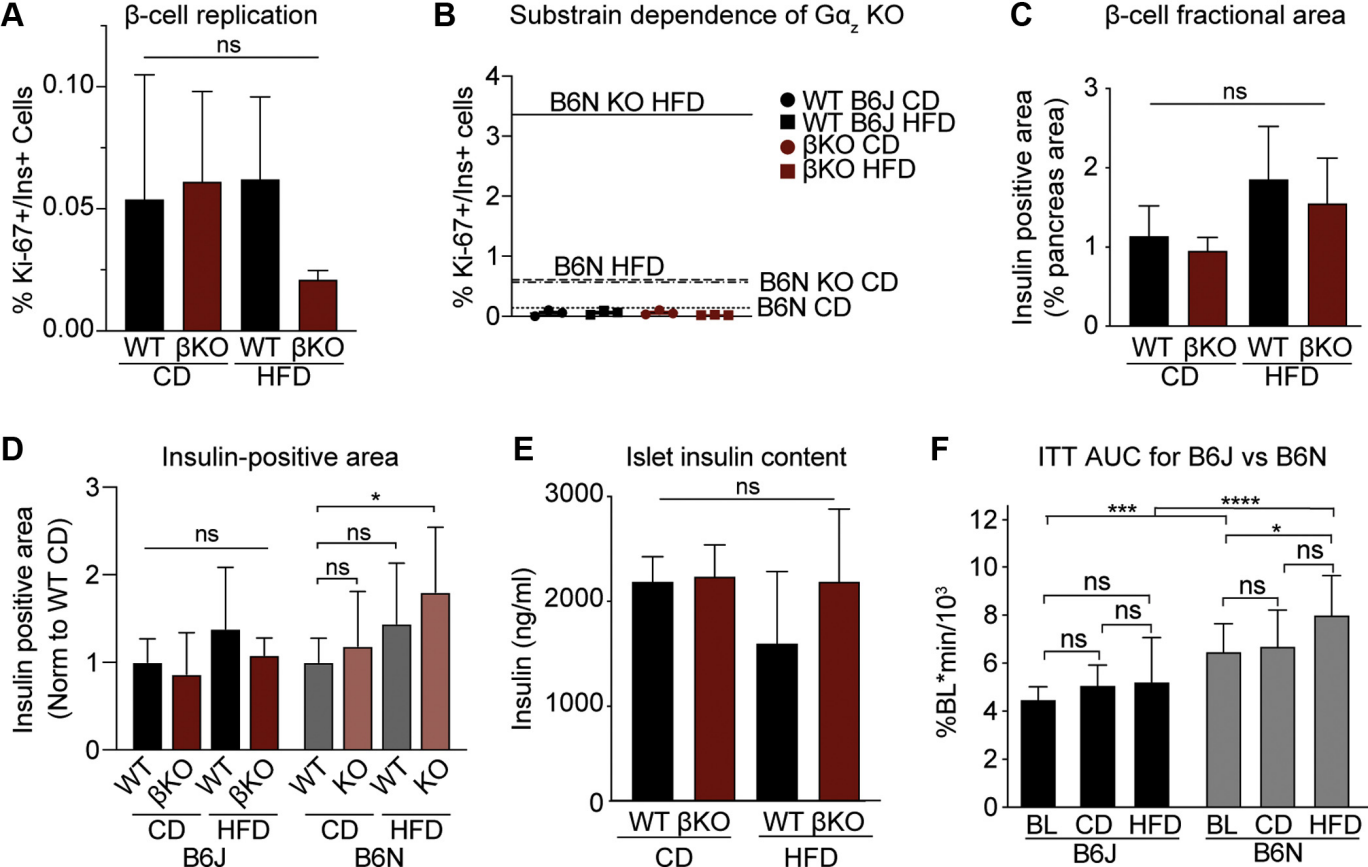
**Beta-cell replication is not affected by HFD feeding or loss of Gα**_**z**_**in insulin-sensitive B6J mice.***A*, quantification of Ki67-positive beta cells by immunofluorescence of pancreas slide sections from WT and βKO mice after CD or HFD feeding. Data represent the percentage total number of beta cells with Ki67-positive fluorescence. All islets from 2 pancreas sections of 3 mice of each group were used in this analysis (n = 3). *B*, raw values for %Ki67-positive beta cells from panel *A* as plotted on the same Y-axis as the original HFD study in full-body Gα_z_ KO mice in B6N background, with the mean values (8) indicated by labeled lines. *C*, the beta-cell fractional area, or the insulin-positive area as a percentage of the total pancreas area from slide sections. Three pancreases/group. *D*, insulin-positive slide area from the 28-week B6J HFD-feeding study described in this work and the previously published 26- to 30-week B6N HFD-feeding study (8), both as normalized to their own WT CD group. N = 3 to 4 pancreas/group. *E*, the insulin content of islets isolated from WT and βKO mice after CD or HFD feeding. N = 3 to 9 independent islet preparations/group. *F*, compiled area under the curve (AUC) analyses of insulin tolerance tests from WT B6J mice (Figs. 1 and 4) and WT B6N mice (Fig. 1 and (8)) at baseline (BL: 11 weeks of age) and after 26 to 30 weeks of CD or HFD feeding. Data represent mean ± SD. Data in panels *A* and *C*–*E* were analyzed by one-way ANOVA with the following preselected comparisons: WT CD *versus* WT HFD, WT CD *versus* KO CD, KO CD *versus* KO HFD, and WT HFD *versus* KO HFD. The Holm–Sidak test was used post hoc to correct for multiple comparisons. In panel *F*, data were analyzed by two-way ANOVA with the Holm–Sidak test post hoc to correct for multiple comparisons. ∗*p* < 0.05, ∗∗∗*p* < 0.01, and ∗∗∗∗*p* < 0.0001. Gα_z_, G protein alpha-subunit; ns, not significant.

Gα_z_ KO mice in the B6N background display a synergistic increase in beta-cell replication with HFD feeding, resulting in significantly increased beta-cell mass (8). Yet, although βKO mice in the B6J background have identical T2D protection as full-body Gα_z_ KO mice in the B6N background, there is no individual or synergistic effect of HFD feeding or Gα_z_ loss on the percentage of Ki67-positive beta cells (Fig. 5*A*). Although the mean Ki67 positivity of HFD-fed βKO beta cells is lower than that of the other three groups, this is not statistically significant (Fig. 5*A*, right), and, when the results are plotted on the same axis as those from the original HFD study in the B6N background (8), none of the data points exceed the mean Ki67 positivity of even CD-fed B6N beta cells (Fig. 5*B*). This phenotypic difference is also apparent in a lack of enhancement of the beta-cell fractional area in B6J mice by Gα_z_ loss or HFD feeding (Fig. 5*C*). Although we did not record pancreas weights in the present study to calculate beta-cell mass, our original study found the greater influence of HFD feeding on beta-cell mass was the islet size/volume (8): a value that may be hidden in beta-cell fractional area calculations if the volume of the pancreas is greater. Therefore, to be able to directly compare the results of our two studies, we normalized the insulin-positive slide area from densiometry analyses to the mean of their own WT control group, revealing the significant enhancement in Gα_z_ KO insulin-positive area by HFD feeding in the B6N substrain is lost in the B6J substrain (Fig. 5*D*). A lack of effect of Gα_z_ loss on beta-cell mass in the B6J substrain is further supported by quantifying islet insulin content, which is unchanged in HFD-fed βKO B6J mice (Fig. 5*E*), as compared with the 2-fold enhancement found in HFD-fed KO B6N mice (8).

Of all of the metabolic phenotypes tested (weight gain, insulin sensitivity, fasting blood glucose, and glucose tolerance), the only phenotype that was significantly different between our two studies was the apparent effect of HFD feeding on insulin sensitivity. Using the blood glucose levels at baseline and 30, 60, and 90 min after insulin challenge—the time points consistent among all of our studies—we calculated insulin areas under the curve (AUCs). Even at baseline (11 weeks of age), B6N mice are much less insulin sensitive than B6J mice, with insulin AUCs approximately one-third greater (Fig. 5*F*). Long-term CD feeding had little effect on insulin AUCs in either substrain, and, while the mean ITT AUC was higher in both HFD-fed groups than their own baseline, this was only statistically significant in the B6N background (Fig. 5*F*). Finally, long-term HFD feeding in the B6N background approximately doubled the ITT AUC, representative of the severe insulin resistance characterized in our previous work (8). These substrain-specific differences are not a factor of beta-cell Cre expression, as insulin sensitivity of Cre-negative littermates was nearly identical (data not shown).

### Beta-cell loss of Gα_z_ improves islet insulin secretion independent of agonist-sensitive EP3 receptor activity

**Figure 6:**
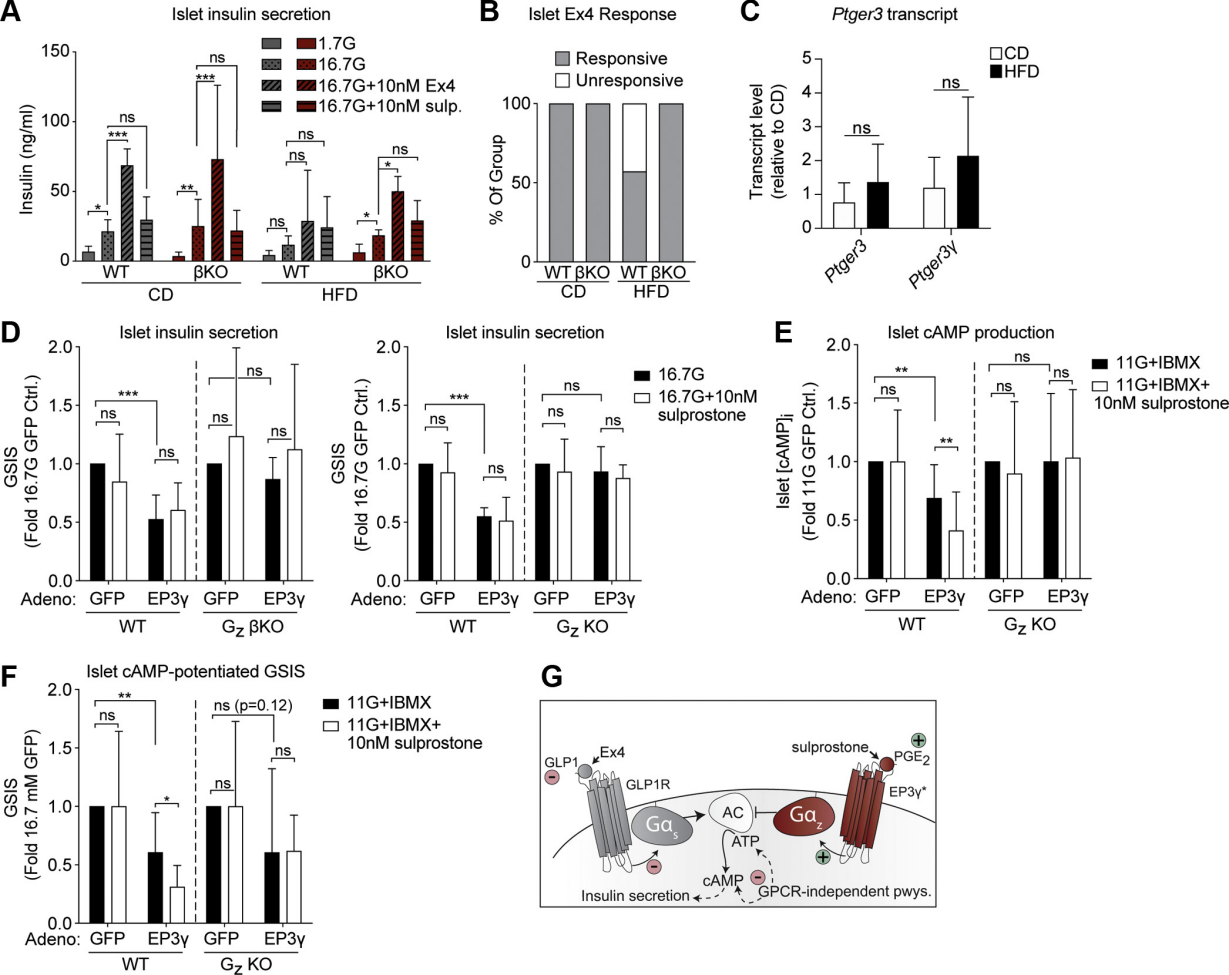
**Loss of beta-cell Gα**_**z**_**preserves islet glucose and incretin responsiveness in HFD-fed mice and ablates the inhibitory effect of EP3γ on islet GSIS and cAMP production.***A*, insulin secreted from WT and βKO mice after 28 weeks of CD or HFD feeding. Islets were treated with the indicated glucose concentrations with or without the addition of exendin-4 (Ex4) or sulprostone. N = 3 to 9 independent islet preparations per group. *B*, the proportion of islet preparations in panel *A* with a higher insulin secretion response with Ex4 than without. *C*, relative islet mRNA expression of the *Ptger3* gene or the EP3γ splice variant (*Ptger3γ*) as measured by qRT-PCR. Cycle times were normalized within each group to beta actin, and the fold change in expression *versus* WT CD calculated *via* the 2^ΔΔCt^ method. N = 4 to 6 independent islet preparations/group. *D*, insulin secreted in stimulatory glucose ± sulprostone in islets isolated from WT and βKO B6J mice (*left*) or WT and KO B6N mice (*right*) when islets are adenovirally overexpressing HA-EP3γ or GFP control. *E*, intracellular cAMP levels ([cAMP]_i_) in islets from GFP- or HA-EP3γ-expressing WT and ΚΟ B6N mice in response to IBMX ± sulprostone. *F*, insulin secreted from the islets shown in panel *E*. In panels *D*–*F*, n = 3 independent islet preparations per group. Data represent mean ± SD and were analyzed as described in Experimental procedures. ∗*p* < 0.05; ∗∗*p* < 0.01; ∗∗∗*p* < 0.001. *G*, the model of the signaling pathways studied in this work and whether they are known to be downregulated (*red minus signs*) or upregulated (*green plus signs*) in the beta-cell dysfunction of T2D. The *asterisk* after EP3γ indicates its partial constitutive activity. B6J, C57BL/6J; B6N, C57BL/6N; CD, control diet; Gα_z_, G protein alpha-subunit; GSIS, glucose-stimulated insulin secretion; HA-EP3γ, hemagglutinin-tagged EP3γ; HFD, high-fat diet; IBMX, 3-isobutyl-1-methylxanthine; ns, not significant; qRT-PCR, quantitative real-time PCR; T2D, type 2 diabetes.

Islets isolated from WT or Gα_z_ βKO mice after 28 weeks of CD or HFD feeding were treated *ex vivo* with basal (1.7 mM) or stimulatory (16.7 mM) concentrations of glucose, the latter with or without 10-nM exendin-4 (Ex4), which acts through the cAMP-stimulatory GLP1R to potentiate GSIS (see model in Fig. 6*G*). Islets from all groups secreted more insulin in response to 16.7-mM glucose *versus* 1.7-mM glucose, although this difference was not statistically significant in islets from WT HFD-fed mice (Fig. 6*A*, solid *versus* dotted bars). Islets from both CD-fed groups had a strong (∼3-fold) potentiating response to 10-nM Ex4, whether shown as the ng/ml secreted insulin (Fig. 6*A*, solid *versus* diagonally striped bars) or the proportion of individual islet preparations secreting more insulin in glucose + Ex4 than glucose alone (Fig. 6*B*). In both cases, islets from WT HFD-fed B6J mice were essentially nonresponsive to Ex4 (Fig. 6, *A*–*B*). Islets from HFD-fed Gα_z_ βKO mice, on the other hand, had a remarkably improved potentiation of GSIS with Ex4 (Fig. 6*A*), and all islet preparations were Ex4 responsive (Fig. 6*B*).

The arachidonic acid metabolite, PGE_2_, is the most abundant native ligand for the cAMP-inhibitory EP3. Circulating PGE metabolite levels are elevated in human subjects with diabetes and in mouse models of the disease, including the extended HFD model used in this work (8, 19, 20, 21, 22, 23, 24, 25). In the beta cell, EP3 has been suggested as specifically coupling to Gα_z_ and not other Gα_i/o_ subfamily members (8, 9, 26, 27, 28) (see model in Fig. 6*G*). To test a role for EP3 in mediating the beta-cell dysfunction observed in the present study, islets were treated with the EP3-selective agonist, sulprostone. Somewhat surprisingly, sulprostone had no effect on GSIS in any group, even in islets from WT HFD-fed mice (Fig. 6*A*, horizontally-striped bars). Furthermore, although *Ptger3* mRNA is expressed in islets from CD- and HFD-fed B6J mice, its abundance does not differ by diet (Fig. 6*C*, left).

The mouse EP3 receptor exists in three splice variants, EP3α, EP3β, and EP3γ, differing in their pharmacological properties (29). Notably, EP3γ has been shown to have up to 80% constitutive activity (30, 31, 32). Splice variant–specific primers for the EP3γ isoform (*Ptger3γ*) confirmed expression in islets from both CD- and HFD-fed B6J mice, again, with no difference by diet (Fig. 6*C*, right). To confirm EP3γ constitutive activity and specific coupling to beta-cell Gα_z_, islets from Gα_z_ βKO B6J mice, Gα_z_ KO B6N mice, or their WT controls were transduced with an adenovirus expressing hemagglutinin (HA)-tagged EP3γ (HA-EP3γ)or GFP control (Fig. S1 confirms HA-EP3γ protein expression by the Western blot). EP3γ expression reduced WT B6J and B6N islet GSIS by about 50%, with no additional effect of sulprostone treatment (Fig. 6*D*). The inhibitory effect of EP3γ was completely ablated in the absence of beta-cell Gα_z_, with no phenotypic difference between islets from full-body Gα_z_ KO or βKO mice, confirming the beta-cell autonomy of this effect (Fig. 6*D*). To confirm specific molecular mechanisms, cAMP production assays were performed in islets from CD-fed WT and KO mice in the presence of the phosphodiesterase inhibitor, 3-isobutyl-1-methylxanthine (IBMX), to block cAMP degradation. These results essentially mirrored those of the GSIS assays, in that EP3γ expression alone had a significant inhibitory impact on [cAMP]_i_ that was dependent on beta-cell Gα_z_ expression (Fig. 6*E*). In [cAMP]_i_ assays, however, sulprostone treatment further reduced [cAMP]_i_ (Fig. 6*E*). These results are not incongruous, as IBMX treatment generates a wide dynamic range for overexpressed EP3γ to reduce cAMP levels, allowing us to detect both constitutive- and agonist-sensitive EP3γ activity, which, in the mouse beta cell, appear solely mediated by Gα_z_. This hypothesis was confirmed by using the stimulation buffer from the cAMP production assay in an insulin ELISA (Fig. 6*F*). Although it is not statistically significant, there may be a cAMP-independent, Gα_z_-independent effect of EP3γ overexpression on GSIS revealed when cAMP levels are clamped high, which we are following up in a different study.

## Discussion

In this work, we confirmed the protection of beta cell–specific Gα_z_ KO mice from HFD-induced hyperglycemia and glucose intolerance nearly exactly phenocopies the protection of full-body Gα_z_ KO mice. The most likely mechanism for this phenotype is improved GSIS, and, more importantly, incretin-potentiated GSIS. Through islet transcriptomics, we found 16-week HFD feeding of WT B6N male mice reduced the expression of a host of genes involved in pancreatic secretion pathways, including those known to be related to cAMP signaling. These gene expression changes occurred early in the disease when fasting blood glucose was only mildly elevated and glucose intolerance had not progressed to fulminant T2D. Although the present study is only correlative, we propose wholesale changes in gene expression patterns, particularly those related to pancreatic secretion and cAMP signaling pathways, may mediate the progression from impaired glucose tolerance to T2D during HFD feeding. Some of the best evidence for the physiological relevance of these alterations in gene expression is that Gα_z_ loss does not just prevent them but actually reverses their directionality. Alternatively, in prior work with the T2D BTBR-Ob mouse model, we find acute treatment with EP3 agonists noncompetitively antagonize agonists of the GLP1R, limiting their maximal potentiating effect on cAMP production and GSIS (22). In the present study, we cannot confirm whether the effects of Gα_z_ loss on enhanced glucose-stimulated and incretin-potentiated insulin secretion are mediated by chronic effects on secretory gene expression profiles, acute modulation of cAMP production, or both, although we favor a combined model.

The acute insulin response after glucose challenge has been well documented to deteriorate with age and duration of HFD feeding in this mouse model of T2D (33, 34, 35). Although islets from HFD-fed Gα_z_ KO mice secrete more insulin in stimulatory glucose than those from WT HFD-fed mice, their GSIS response is not returned to that of CD-fed animals, regardless of whether mice are in the B6N or B6J background (8). These *ex vivo* islet results are consistent with those of *in vivo* oral glucose tolerance tests (OGTTs), as after 16 weeks or 28 weeks of HFD feeding, the blood glucose levels of Gα_z_ KO or βKO mice are just as elevated as their WT HFD-fed counterparts. Long-term HFD feeding of mice in B6 substrains functionally uncouples voltage-dependent calcium channels from insulin secretory granules (36). This effect is reproduced by chronic treatment of primary mouse or human islets with high levels of palmitate: a mimic of the overnutrition, inflammation, and dyslipidemia associated with obesity and insulin resistance (37). In light of this, it is not surprising that Gα_z_ loss does not fully restore GSIS to that of islets from CD-fed mice.

By 30 min after oral glucose challenge, the blood glucose levels of HFD-fed Gα_z_ KO and βKO mice are indistinguishable from those of CD-fed mice. A defective incretin response has long been understood as a primary contributor to the beta-cell dysfunction of T2D (38, 39). In the present study, an improved *ex vivo* incretin response is readily apparent in islets from Gα_z_ βKO mice as compared with WT HFD-fed controls. As we did not measure plasma insulin levels during OGTTs, we cannot confirm this correlates with increased incretin-potentiated release of insulin from beta cells *in vivo*. Yet, particularly in HFD feeding models, plasma insulin levels are a poor correlate of beta-cell function (33, 34). As compared with B6N mice, 16-week HFD-fed B6J mice have only a mild potentiation of plasma insulin after glucose challenge, and, in both substrains, this potentiation has returned to baseline by 5 to 10 min (18). This is consistent with our original HFD study, in which only a weak elevation in plasma insulin levels was discovered 10 min after glucose administration in HFD-fed B6N mice (8). In a lean, healthy background, however, the potentiation of *ex vivo* islet and *in vivo* GSIS responses by Gα_z_ loss do directly correlate with each other, as evidenced by studies of Gα_z_ KO mice in the Balb/c strain (7).

Previous findings by our group showed full-body loss of Gα_z_ results significantly augments beta-cell proliferation and beta-cell mass during HFD feeding (8). In this study, we did not observe a beta-cell autonomous role for Gα_z_ in beta-cell proliferation. This was somewhat surprising, as in the B6N study, Gα_z_ loss alone augments beta-cell Ki67 positivity after 30 weeks of HFD feeding, although not nearly to the extent as when Gα_z_ KO mice are fed the HFD (8). Furthermore, EP3 has been identified as a negative regulator of beta-cell proliferation (12). We believe this phenotypic discrepancy is attributable to B6 substrain differences that affect their metabolic phenotype (40). Young B6N mice start out with some degree of insulin resistance: a phenotype that is affected by HFD feeding to a much greater degree than in the B6J substrain (18, 40). As cAMP signaling has been shown to be important in the beta-cell proliferative response (41), loss of beta-cell Gα_z_ may be permissive toward enhanced replication in context where beta-cell mass is required, such as in the pathophysiological context of moderate-to-severe insulin resistance. On the other hand, known cell-cycle genes were poorly represented among the top 100+ genes differentially expressed in our transcriptomics analysis. Furthermore, when tested by qRT-PCR, their expression was actually lower in islets from KO HFD-fed mice than in WT HFD-fed mice. Therefore, it is also possible the enhanced secretory phenotype of HFD-fed KO mice, allowing them to effectively maintain euglycemia long-term, prevents the beta-cell stress that would detrimentally affect beta-cell replication. In this model, enhanced beta-cell replication would be completely Gα_z_ independent. Future studies will be required to differentiate these possibilities.

Four decades ago, elevated PGE_2_ signaling was postulated as contributing to the beta-cell dysfunction of diabetes (23, 24, 25, 42). Previous work from our group confirmed the role agonist-sensitive EP3 signaling in the beta-cell dysfunction of T2D, both with regard to cAMP production and downstream GSIS (8, 22, 43). Figure 6*G* shows our model of the GPCR-mediated signaling pathways studied in this work and how, independent of any changes in EP3 expression, loss of Gα_z_ can compensate for the underlying secretory defects found in mouse models of T2D. EP3γ, although it is agonist responsive, has robust constitutive activity. Therefore, because beta-cell EP3γ is solely coupled to Gα_z_, loss of Gα_z_ ablates the ability of EP3γ to act as a tonic brake on cAMP production, reducing the ability of the beta cell to secrete insulin in response to glucose, but, more importantly, the ability of GLP1R agonists to potentiate this response. Although there is no increase in EP3γ expression in B6J islets with HFD feeding, the brake EP3γ elicits is likely stronger in the context of HFD feeding, where circulating PGE_2_ levels are known to be elevated (8). In the right context, chronic loss of EP3γ activity may be permissive toward increased beta-cell mass, although effects on secretion alone appear sufficient to explain the phenotype observed in the 28-week HFD study performed in this work. Continued study of this previously undescribed agonist-independent EP3γ/Gα_z_ signaling pathway may help in identifying new T2D therapies, as well as improving the efficacy of cAMP-based therapies currently being used.

## Experimental procedures

### Antibodies and reagents

Radioimmunoassay (RIA)-grade bovine serum albumin (A7888) and Ex4 (E7144) were purchased from Sigma-Aldrich. Insulin ELISA antibodies (insulin + proinsulin antibody, 10R-I136a; insulin + proinsulin antibody, biotinylated, 61E-I136bBT) were from Fitzgerald Industries. The 10 ng/ml insulin standard (8013-K) and assay buffer (AB-PHK) were from Millipore. RPMI 1640 medium (11879–020, no glucose), penicillin/streptomycin (15070–063), and fetal bovine serum (12306C: qualified, heat inactivated, USDA-approved regions) were from Life Technologies. Dextrose (D14–500) was from Fisher Scientific. Mouse anti-actin (8H10D10), goat anti-rabbit horseradish peroxidase (HRP)-conjugated secondary antibody (7074), and horse anti-mouse HRP-conjugated secondary antibody (7076) were from Cell Signaling Technology. Guinea pig anti-insulin (discontinued), background-reducing antibody diluent (S302281-2), and serum-free protein block (X090930-2) were from Dako (Agilent). A second guinea pig anti-insulin was from Abcam (AB7842). Rabbit anti-Ki67 mAb D3B5 (9129) was from Cell Signaling Technology. Citrate-based antigen-retrieval solution (H-3300) and VECTASHIELD Mounting Medium with DAPI (H-2000) were from Vector Laboratories. FITC-coupled donkey anti-rabbit antibody (711-095-152) and Cy3-coupled goat anti–guinea pig antibody (707-165-148) were from Jackson Immunoresearch. The RNeasy Mini Kit (74104) and RNase-free DNase set (79254) were from Qiagen. High-Capacity cDNA Reverse Transcription Kit (4368814) was from Applied Biosystems. Rat anti-HA High-Affinity antibody (11867423001) and FastStart Universal SYBR Green Master Mix (4913850001) were from Millipore-Sigma. Sulprostone (14765) was from Cayman Chemical. Pierce ECL plus (32132) and the Pierce bicinchoninic acid (BCA) Protein Assay Kit (23227) were from Thermo Fisher.

### Animal husbandry and the diet-induced obesity model

Gα_z_ full-body KO mice on the B6N background (FBKO) and βKO on the B6J background have both been previously described and validated for the expected tissue-specific loss of Gα_z_ protein (6, 8). Breeding colonies were maintained at the University of Wisconsin Breeding Core Facility. Experimental mice were generated by single or double heterozygous crosses as appropriate and transferred to investigator-accessible housing in the William S. Middleton Memorial Veterans Hospital Animal Research Facility between 3 and 4 weeks of age. Mice were maintained in a temperature- and humidity-controlled environment with a 12:12-h light/dark cycle and *ad libitum* access to pelleted mouse chow (Rodent Laboratory Chow 5001; Purina, Neenah, WI) and acidified water (Innovive, San Diego, CA). At 11 weeks of age, experimental mice were switched to either a 45 kcal% fat diet (D12451; Research Diets, New Brunswick, NJ) or 10 kcal% fat diet (D12450B; Research Diets), both irradiated, for either 16 weeks (FBKO microarray study) or 28 weeks (βKO phenotyping study). These studies were approved by the Institutional Animal Care and Use Committees of the University of Wisconsin-Madison and the William S. Middleton Memorial Veterans Hospital, which are both accredited by the Association for Assessment and Accreditation of Laboratory Animal Care. Treatment of all animals was per the standards set forth by the National Institutes of Health Office of Animal Care and Use. Only male mice were used in these experiments.

### OGTTs and ITTs

On the morning of testing, mice were weighed and transferred to new cages free of food for 4 to 6 h. Blood glucose was recorded by tail nick using an AlphaTRAK veterinary glucometer and rat/mouse-specific test strips (Zoetis, Parsippany, NJ). OGTTs were performed by gavage of a sterile 1 g/kg glucose solution in 0.9% saline (made using a 20% glucose stock), and ITTs were performed by intraperitoneal injection of 0.75 U/kg short-acting recombinant human insulin (Humulin R; Eli Lilly). Blood glucose readings were averaged within genotypes at each time point and data presented as means ± SE (OGTT) or percentage baseline blood glucose (ITT). AUC analyses were calculated by normalizing data from each mouse to its own baseline value. OGTT and ITT glucose excursion curves were analyzed by two-way paired ANOVA with row means for each time point compared with those of WT CD-fed mice and the Holm–Sidak test post hoc to correct for multiple comparisons. Final body weights, final fasting blood glucose levels, and OGTT and ITT AUC results were analyzed by one-way ANOVA of the following preselected comparisons: WT CD *versus* WT HFD, WT CD *versus* KO CD, KO CD *versus* KO HFD, and WT HFD *versus* KO HFD. The Holm–Sidak test was used post hoc to correct for multiple comparisons.

### Immunohistochemistry and immunofluorescence analyses of pancreas slide sections

Insulin immunohistochemistry and insulin and Ki67 immunofluorescence were performed on formalin-fixed, paraffin-embedded pancreas sections essentially as previously described (5, 8). Briefly, for insulin insulin immunohistochemistry, a 1:500 dilution of Dako guinea pig anti-insulin primary antibody and 1:2000 goat anti-guinea pig HRP-coupled secondary antibody were used. After development, slides were counter-stained with hematoxylin and imaged using an EVOS FL automated pan-and-stitch microscope (Thermo Fisher). Images were analyzed using ImageJ (64-bit) software (the National Institutes of Health, Bethesda, MD) with shading correction to quantify the total insulin-positive area (representative of the islet size) and the insulin-positive area as normalized to the total area of the pancreas section (beta-cell fractional area). Pancreas sections from N = 3 mice per group were used in this analysis.

For insulin and Ki67 coimmunofluorescence experiments, rabbit anti-Ki67 (1:200) and guinea pig anti-insulin (Abcam - 1:75) were applied to sections overnight at 4 °C. After a PBS wash, slides were incubated for 30 min with 1:400 FITC anti-rabbit and 1:200 Cy3 anti–guinea pig secondary. Sections were counterstained with DAPI using the VECTASHIELD Mounting Medium. Images were captured using uniform exposure settings on a Leica DM4000B microscope (Leica Microsystems, Wetzlar, Germany) and photographed with a Retiga 4000R digital camera (QImaging Systems, Surrey, BC, Canada). ImageJ was used to manually count Ki67-positive and insulin-positive nuclei, and the percentage of Ki67+ beta cells for each islet was calculated. All islets from the entire pancreas section area were quantified, with two sections quantified per mouse (N = 3 mice/group).

### Mouse pancreatic islet isolation and culture

At the indicated ages before or after control or HFD feeding, islets were isolated from experimental mice using a collagenase digestion procedure as described (44). In some cases, islets were cultured in RPMI 1640 containing the indicated glucose concentration and 10% heat-inactivated FBS and penicillin/streptomycin before downstream assay.

### RNA isolation and quality control for microarray experiments

Approximately 300 islets per mouse (N = 5 per group) were hand-picked from contaminating acinar tissue into a microfuge tube containing 1-ml PBS and washed one additional time with PBS, using a pulse vortex/centrifugation method previously described. PBS was gently aspirated from the islet pellet, and 200-μl RLT buffer (Qiagen) was added. Islets were lysed using a plastic micropestle homogenizer, and RNA was purified using the RNeasy Mini Kit (Qiagen) according to the manufacturer’s directions. RNA quality was assessed by electrophoresis using a Eukaryote Total RNA Pico cartridge and Bioanalyzer 2100 instrument (Agilent). The minimum RNA Integrity Score of samples subject to microarray analysis was 7.40, with most samples having RNA Integrity Scores of 8.0 or above. One HFD-fed WT mouse islet sample and one HFD-fed Gα_z_ KO islet sample failed RNA quality control.

### Islet exon array and quantitative PCR gene expression analyses

Islet gene expression patterns were quantified using Mouse GeneChip Exon 1.0 ST Array chips (Affymetrix) by the University of Wisconsin Biotechnology Center, Gene Expression Center. The output data were analyzed using R, version 3.5.3 (45). Differential gene expression was quantified, and figures were produced using the limma package with the metabolomics wraparound package (46). Genes without an ID were removed from analysis; therefore, our analysis was based on 16,291 genes. Our final analysis represents independent islets preparations from N = 5 WT CD-fed mice, N = 4 WT HFD-fed mice, N = 5 Gα_z_ KO CD-fed mice, and N = 3 Gα_z_ KO HFD-fed mice, as data from one Gα_z_ KO HFD-fed mouse islet sample was removed from further analysis because of anomalies identified during data exploration. To detect differentially expressed genes between treatment groups, an empirical Bayes moderated linear model was fitted to each gene (47). The empirical Bayes approach shrinks the estimated sample variances by borrowing information from across genes. Fold changes were estimated using contrasts between WT CD and WT HFD mice, WT CD and KO CD, and WT HFD and KO HFD mice. *p*-values for each comparison were adjusted using the Benjamini–Hochberg procedure using a false discovery rate of 5% (48). Testing for over-representation of KEGG pathways was performed using genes with an adjusted *p*-value from the linear model of <0.05 (49). KEGG pathways were considered with an adjusted *p*-value of <0.05. A principal component analysis plot was performed on all identified genes using the limma metabolomics package. For a selected subset of genes, microarray results were confirmed by relative qRT-PCR using cDNA generated from purified RNA samples and SYBR Green Master Mix using a previously established protocol (5).

### Islet insulin and glucagon secretion assays from 26- to 30-week FBKO HFD feeding study

Islets from HFD-fed WT or Gα_z_ KO mice were cultured overnight in an islet medium containing 8.4-mM glucose before assay. For static insulin secretion assays, islets were washed and preincubated in Krebs-Ringer bicarbonate buffer (KRBB) containing 2.8-mM glucose for 45 min before being switched to 12-mM glucose for 45 min. For static glucagon secretion assays, islets were washed and preincubated in KRBB containing 25-mM glucose for 45 min before being switched to 2.8-mM glucose for 45 min. The secretion buffer was decanted, islets were washed in PBS and resuspended in 100 μl of lysis buffer (20-mM Tris-HCl, pH 7.5; 150-mM NaCl, 1-mM Na_2_EDTA, 1-mM EGTA, 1% Triton X, 2.5-mM sodium pyrophosphate, 1-mM β-glycerophosphate, 1-mM sodium orthovanadate, and 1 μg/ml leupeptin), and the BCA assay was performed to determine the protein content. Secreted insulin and glucagon were measured by RIA according to the manufacturers’ instructions (Coat-A-Count Insulin RIA kit, Diagnostic Products Corp, Los Angeles, CA, and Glucagon RIA kit, Linco Research, St Louis, MO). Secreted insulin (μIU) and secreted glucagon (pg/ml) were both normalized to the total protein content (in μg) of the corresponding islet pellet. Other phenotyping results have been previously published (8).

### Islet insulin secretion assays from 28-week βKO HFD feeding study

Islets were plated individually into a 96-well V-bottomed tissue culture-treated plate containing 100 μl of islet medium per well and incubated overnight in a tissue-culture incubator, during which time fibroblasts surrounding the islet adhere to the dish. Single-islet GSIS assays were performed essentially as previously described (50). Briefly, islets were washed and preincubated for 45 min in a 0.5% BSA KRBB containing 1.7-mM glucose and 0.5% BSA. Islets were then incubated for an additional 45 min in the buffer containing the indicated glucose concentrations and compounds. The secretion medium was collected, and islets were washed 1X in PBS and lysed in 100 μl of lysis buffer to determine the insulin content. Insulin was assayed by in-house ELISA as previously described (22). To account for islet loss during the assay, replicates with insulin content values <200 ng/ml (∼10-fold less than the mean of all other replicates) or percent insulin secretion values above 10% (∼10-fold higher than the mean of all other replicates) were excluded from the analysis. The group with the most islet loss was the WT-HFD group, with approximately 12.5% of islets disadhering (a result consistent with our observations of adherence being affected by islet health). Islets from the other 3 groups had little-to-no islet loss during the assay.

### Adenoviral transduction of primary islets for exogenous protein expression

To create an adenovirus-expressing mouse EP3γ, the coding sequence of an N-terminally HA-EP3γ was amplified from a pcDNA3.1 vector (a kind gift of Dr Richard Breyer, Vanderbilt University) and cloned in pVQAd CMV eGFP (ViraQuest, North Liberty, IN) using a Gibson assembly kit according to the manufacturer’s protocol (New England BioLabs). The resulting construct was sequence-validated by the UW Biotechnology Center before being shipped to ViraQuest (North Liberty, IN) for virus amplification, purification, concentration, validation of replication incompetence, and quantification of plaque-forming units (PFU). High-titer VQAd CMV eGFP HA-EP3γ adenovirus (∼10^12^ PFU) was shipped back to the investigator, along with a high-titer VQAd CMV eGFP control. To overexpress GFP or HA-EP3γ in primary mouse islets, freshly isolated islets were picked into a 6-cm petri dish containing 0.5 μl/ml high-titer adenovirus for 2 to 3 h before being transferred to a dish containing a fresh islet medium. All downstream assays were performed 3 days after transduction. Adenovirally transduced islets used for the Western blot and GSIS assays were from 10- to 12-week-old chow-fed Gα_z_ FBKO or βKO mice or their WT controls, and islets used in cAMP assays were from 34- to 42-week-old WT and Gα_z_ βKO mice fed the 10 kcal% fat CD for 22 to 30 weeks starting at 12 weeks of age.

### Western blot

For each lane, approximately 150 islets, 3 days after transduction, were pelleted, washed, resuspended in 100-μl lysis buffer and sonicated 3 times for 10 s to ensure islets were fully disrupted. The protein content was determined by the BCA assay. Approximately 30-μg protein per sample was subjected to SDS/PAGE and transferred to polyvinylidene fluoride membranes, and immunoblotting was performed with rat anti-HA (1:1000 dilution) or mouse anti–β-actin (1:1000 dilution) according to standard protocols. Molecular weight markers were a 1:1 mix of Magic Mark XP (Thermo Fisher) for chemiluminescent detection and Precision Plus Dual Color (Bio-Rad) for visual detection. The membrane was stripped in-between the Western blot for HA and β-actin using an acidic glycine stripping buffer (0.1 M glycine, 20-mM magnesium acetate, 50-mM KCl, pH 2.2). Proteins were detected by enhanced chemiluminescence.

### cAMP production assay and modified GSIS protocol for adenovirally transduced islets

Adenovirally transduced islets were subject to assay for intracellular cAMP production using a previously described protocol (44). Briefly, on the day of assay, islets were hand-picked from RPMI into a dish containing 3-mM glucose KRBB and then 15 to 20 islets per replicate picked into microfuge tubes containing 1 ml of the same buffer. Tubes were incubated uncapped in a 37 °C tissue culture incubator for 45 min (before incubation), followed by a buffer switch into 11-mM glucose KRBB with 200-μM IBMX with or without the addition of 10-nM sulprostone and a second 45-min incubation (stimulation). Islets were pulse vortexed/centrifuged, stimulation buffer decanted, islets washed in PBS, and pellets flash-frozen in liquid nitrogen and stored at −80 °C until the day of the assay. The cellular cAMP content was analyzed using the Amersham cAMP Biotrak Enzyme Immunoassay (EIA) System (GE Healthcare Life Sciences) using the nonacetylation procedure with novel lysis reagents according to the manufacturer's instructions. Three mice were used per genotype, triplicate biological replicates were performed for each virus/treatment per islet preparation, and two technical EIA replicates were performed and averaged before data analysis. To normalize for the islet volume, the residual islet lysates remaining after the cAMP EIA plates that had been loaded (∼10 μl) were used in our in-house insulin ELISA to determine the insulin content. Four of 72 sample wells had “OVERFLOW” readings, meaning that their corresponding [cAMP]_i_ values could not be normalized and were excluded from further analysis. Static GSIS assays were performed on separate islet preparations using essentially the same protocol except for the addition of IBMX to the stimulation buffer. Biological replicates were assayed in triplicate on our in-house insulin ELISA, and triplicates were averaged to give the final insulin content and secreted insulin values. As with the cAMP assay, secretion data were normalized within each experiment to the value observed in GFP-expressing islets treated with glucose alone. In both assays, raw data for each islet preparation were normalized to the [cAMP]_i_ or GSIS of GFP-expressing islets in glucose alone.

### Statistical analyses

All data, excluding the microarray data, were analyzed using GraphPad Prism, v. 8 (GraphPad Software Inc, San Diego, CA). Unless otherwise specified, single comparisons between two groups were performed by two-way T-test, and group analyses were performed by one-way ANOVA or two-way ANOVA, paired or unpaired as appropriate, all with Holm-Sidak test post hoc to correct for multiple comparisons. For normalized cAMP and GSIS data, which did not have a consistent SD, the effects of EP3γ expression and/or sulprostone treatment were analyzed by multiple T-test with fewer assumptions, followed by the Holm-Sidak test post hoc to correct for multiple comparisons. In all cases, *p* < 0.05 was considered statistically significant.

## Data availability

Raw mouse exon data have been deposited with the NCBI gene expression omnibus (GEO) as accession ID GSE154325. All other data are contained within the manuscript and available on request.

## Conflict of interest

M. D. S., C. L. G., S. J. G., E. G., K. A. C., J. M. H., G. M. K., A. R., M. L. W., and M. E. K. declare that they have no conflicts of interest with the contents of this article. J. C. N. is currently a Novo Nordisk Inc employee (800 Scudders Mill Road, Plainsboro, NJ 08536). A. L. B. is currently a Pfizer employee (235 East 42nd Street, New York, NY 10017). This work was completed in full during their post-baccalaureate and/or predoctoral training with Dr Kimple and are not related to their current positions. D. W. L. has received funding from, and is a scientific advisory board member of, Aeovian Pharmaceuticals, which seeks to develop novel, selective mTOR inhibitors for the treatment of various diseases, including diabetes. At present, there are no data to support relevance of mTOR inhibitors to the work described in this article.
